# A base promoted one pot solvent free version of the Ramachary reductive coupling/alkylation reaction for the synthesis of 2,2-disubstituted ethyl cyanoacetates†

**DOI:** 10.1039/c8ra00326b

**Published:** 2018-02-28

**Authors:** Guangyou Jiang, Min Liu, Dongmei Fang, Ping Tan, Min Huang, Taiping Zhou, Zhenju Jiang, Zhihong Xu, Zhouyu Wang

**Affiliations:** Department of Chemistry, Xihua University Chengdu 610039 China zhouyuwang77@163.com xzh1966@163.com +86-028-8772-3006 +86-028-8772-9463; Chengdu Institute of Biology, Chinese Academy of Sciences Chengdu 610041 China

## Abstract

An *N*,*N*-diisopropylethylamine promoted solvent-free Ramachary reductive coupling/alkylation (RRC/A) reaction for the synthesis of 2,2-disubstituted ethyl cyanoacetates has been developed. A series of 2,2-disubstituted ethyl cyanoacetates were synthesized in one pot by the RRC/A reaction of commercially available aldehydes, ethyl cyanacetates, alkyl halides and Hantzsch ester. A solvent free two step multicomponent reaction has also been developed for the preparation of 2,2-dialkylated malononitriles and 2,2-dialkylated 4-nitrophenyl acetonitriles. All the designed RRC/A products could be easily obtained with good yields by these methods.

## Abstract 2

An *N*,*N*-diisopropylethylamine promoted solvent-free Ramachary reductive coupling/alkylation (RRC/A) reaction for the synthesis of 2,2-disubstituted ethyl cyanoacetates has been developed.

## Graphical abstract

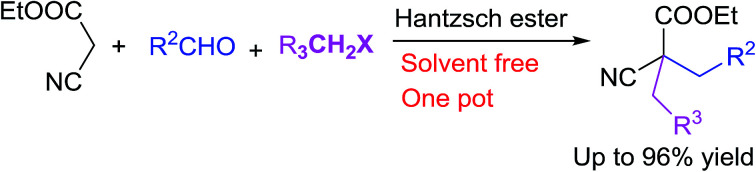

Cyanoalkyl moieties are found as important structural motifs in several nitrile-containing natural products and drugs. Nitrile-containing compounds are also important synthons in organic synthesis because the cyano group can be easily converted into other functional groups (Fig. 1). Direct alkylation of simple alkylnitriles normally requires the utilization of strong bases, which are usually incompatible with base sensitive substrates.^1–5^ Thus, the activated nitriles such as α-cyano esters, malononitriles were frequently used as substrate in the mild base catalyzed alkylation reactions.^6^ (Scheme 1).

**Fig. 1:**
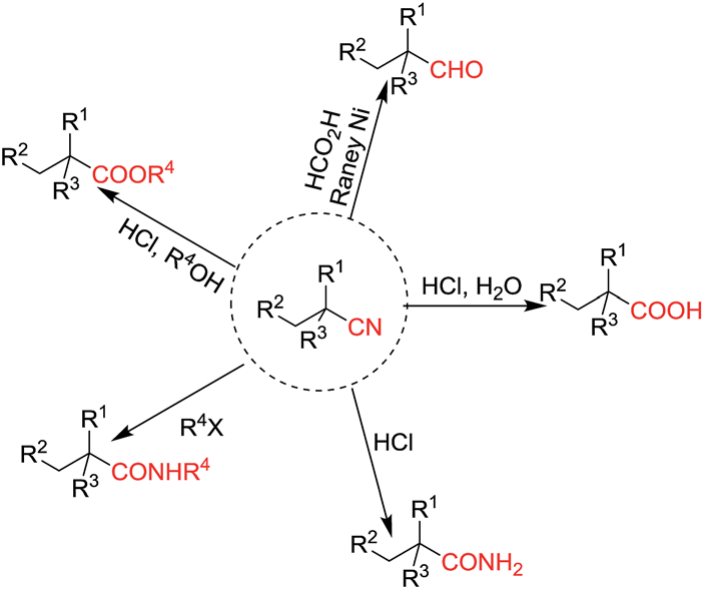
Transformations of nitriles.

**Scheme 1:**
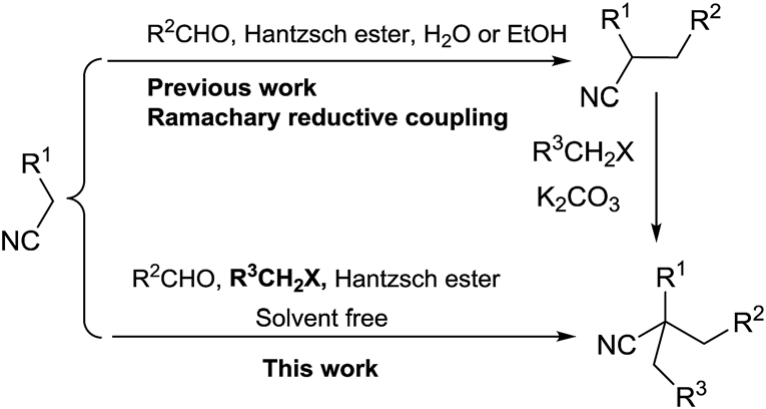
Methods for the preparation of 2,2,2-trisubstituted nitriles.

During the last decade, there have been considerable advances in the organocatalysts catalyzed direct tandem reactions of activated nitriles.^7–14^ In 2006, Ramachary and coworkers reported the first l-proline catalyzed Knoevenagel/hydrogenation (K/H) and Knoevenagel/hydrogenation/alkylation (K/H/A) for the preparation of substituted nitrile-containing products.^13,14^ The Ramachary reductive coupling (RRC) and Ramachary reductive coupling/alkalation (RRC/A) process could be done smoothly in EtOH at room temperature under the catalysis of l-proline to get the 2-alkylated cyano esters. In addition, lots of pharmaceutical intermediates and functionalized molecules were synthesized by the RRC and RRC/A reactions.^15–24^ Following this pioneering RRC reaction, we reported the first base-promoted RRC process in water for the preparation of a series of nitrile-containing products.^25^ The method provided convenient ways for the construction of nitrile-containing compounds in which the cyano group is attached to a tertiary carbon atom. For the construction of nitrile compounds in which the cyano group is attached to a quaternary carbon atom, Ramachary and coworkers used the RRC/A reaction. However, the alkylation reagents should be added after the completion of the RRC process.^13,14^ As part of our program to develop practical method for the construction of pharmaceutical products,^25–29^ here in, we report the first solvent free one-pot four components reaction for the preparation of 2,2-dialkylated cyano esters from commercially available aldehydes, ethyl cyanacetates, alkyl halides and Hantzsch ester as shown in Scheme 1.

At the onset of our study, malononitrile, 4-nitrophenyl acetonitrile, and ethyl cyanacetate were used, respectively, as model substrate in the reaction. When nitrile, 4-bromobenzaldehyde, benzyl bromide and Hantzsch ester were heated to 80 °C, without the addition of base, no RRC/A product could be detected for all the tested nitriles in our study. When 2 equivalents of sodium bicarbonate were added in the reaction, the RRC/A product with 40% yield could isolated from the reaction in which the ethyl cyanacetate was used as starting material. For the reaction in which malononitrile and 4-nitrophenyl acetonitrile were used as starting material, respectively, the direct alkylation of these nitriles by the benzyl bromide were found as the major product under the otherwise identical reaction conditions. Thus ethyl cyanacetate was chosen as the model substrate in the further studies (Table 1).

**Table 1:** Base promoted Ramachary reductive coupling/alkylation reaction^a^

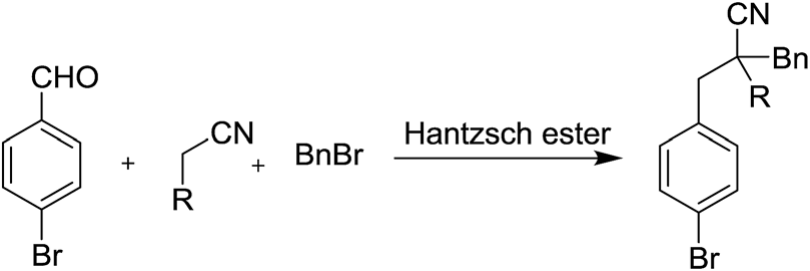				
Entry	R	Additives (eq.)	*T* (°C)	Yield^b^ (%)
1	CN	—	80	—
2	CN	NaHCO_3_ (2.0)	80	—
3	4-NO_2_C_6_H_4_	—	80	—
4	4-NO_2_C_6_H_4_	NaHCO_3_ (2.0)	80	—
5	COOEt	—	80	—
6		NaHCO_3_ (2.0)	80	40
7		Na_2_CO_3_ (2.0)	80	60
8		K_2_CO_3_ (2.0)	80	76
9		NaOH (2.0)	80	5
10		TEOA (2.0)	80	30
11		DEAE (2.0)	80	40
12		DABCO (2.0)	80	20
13		DIEA (2.0)	80	90
14		DIEA (1.0)	80	50
15		DIEA (3.0)	80	88
16		DIEA (4.0)	80	43
17		DIEA (2.0)	60	—
18		DIEA (2.0)	70	—
19		DIEA (2.0)	90	92
20^c^		DIEA (2.0)	90	67
21^d^		DIEA (2.0)	90	73
22^e^		DIEA (2.0)	90	92

a 0.25 mmol of 4-bromobenzaldehyde, 0.75 mmol of BnBr, 0.30 mmol of Hantzsh ester and 0.30 mmol of nitrile were added in reaction tube, and heating for 12 h.

b Isolated yield.

c Reaction time 0.5 h.

d Reaction time 1 h.

e Reaction time 2 h. TEOA = triethanolamine; DEAE = 2-diethylamino ethanol; DABCO = triethylenediamine. DIEA = *N*,*N*-diisopropylethylamine.

Next we found the yield of the RRC/A product could be increased to 60% and 76%, when 2 equivalents of sodium carbonate and potassium carbonate were added in the reaction, respectively. However, the yield of the RRC/A product was dropped to 5% when 2 equivalents of sodium hydroxide were added in the reaction. No more than 50% yield was obtained when organic base such as triethanolamine, 2-diethylamino ethanol and triethylenediamine were added in the reaction, respectively. Fortunately, we found 90% yield could be achieved when 2 equivalents of *N*,*N*-diisopropylethylamine (DIEA) were added under the otherwise identical reaction conditions. Decreasing the amount of DIEA to 1 equivalent, the yield of the RRC/A product was dropped to 50%. However, increasing the amount of DIEA to 3 and 4 equivalents, respectively, a low yield was also observed. Only trace amount of the RRC/A product was detected when the reactions were set up at 60 °C and 70 °C, respectively. Increasing the reaction temperature to 90 °C, the yield of the RRC/A product could be increased to 92%. When the reaction was heated at 90 °C for 2 hours, 92% yield of the RRC/A product was observed. However, only 67% and 75% yields were obtained when the reaction time was cut down to 0.5 and 1 hour, respectively. After careful investigation, we identified the best reaction conditions in which the ethyl cyanacetate, 4-bromobenzaldehyde, benzyl bromide and Hantzsch ester were heated at 90 °C for 2 h, and the RRC/A product could be obtained with 92% isolated yield.

With the optimized reaction conditions in hand, the substrate scope and limitations of the solvent-free one-pot four components reaction was studied (Fig. 2). We found, beside 4-bromobenzaldehyde, other halide substituted benzaldehydes such as 4-cholorobenzaldehyde and 4-flurobenzaldehyde could also be used as good substrate in the reaction, and the RRC/A products 1b and 1c could be obtained with 95% and 96% yield when they were used in the reaction, respectively. The product 1d could be obtained with 94% yield when 4-methyl benzaldehyde was used in the reaction. The yield of RRC/A product 1e was dropped to 73% when 4-methoxyl benzaldehyde was used. And the yield of 1f was further dropped to 24% when 4-cyano benzaldehyde was used in the reaction. However, when the 4-nitro benzaldehyde was used in the current reaction, the RRC/A product 1g could be obtained with 76% yield. 1-Naphthaldehyde could also be used in the reaction and the RRC/A product 1h was obtained with 92% yield. Other aldehydes such as furan-2-carbaldehyde and thiophene-2-carbaldehyde could also be used in the reaction, and the RRC/A product 1i and 1j was isolated with 94% and 88% yields, respectively. No reduction RRC/A product could be obtained when acetophenone was used in the reaction. And aliphatic aldehydes such as propionaldehyde was not good substrate, either. The low boiling point of propionaldehyde and lower reactivity of ketone may be responsible for it.

**Fig. 2:**
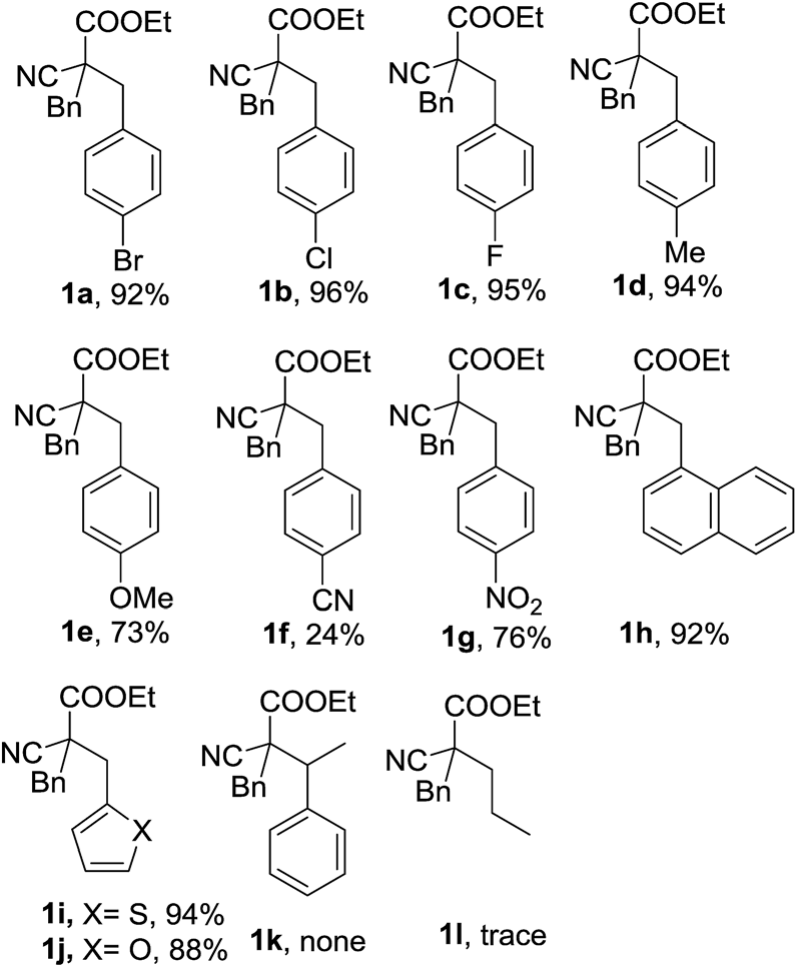
Substrate scope of the solvent-free one-pot four components reaction.

For the preparation of 2,2-dialkylated malononitriles and 2,2-dialkylated 4-nitrophenyl acetonitriles, a base promoted one-pot two step RRC/A process was also developed. However, a self-catalyzed RRC process was observed in the reaction of malononitrile, aldehyde and Hantzsch ester. The RRC product 2-(4-bromobenzyl) malononitrile could be obtained with 95% yield under the optimized reaction conditions. After the completion of the self-catalyzed RRC process, without any work up process, the benzyl bromide with DIEA were added, and then the RRC/A product 2 was obtained with 93% isolated yield. In the preparation of RRC/A product 3, both the RRC process and alkylation step need one equivalent of *N*,*N*-diisopropylethylamine. A series of derivatives of the nitrile compounds could be prepared by these two methods (for more details see ESI†) (Scheme 2).

**Scheme 2:**
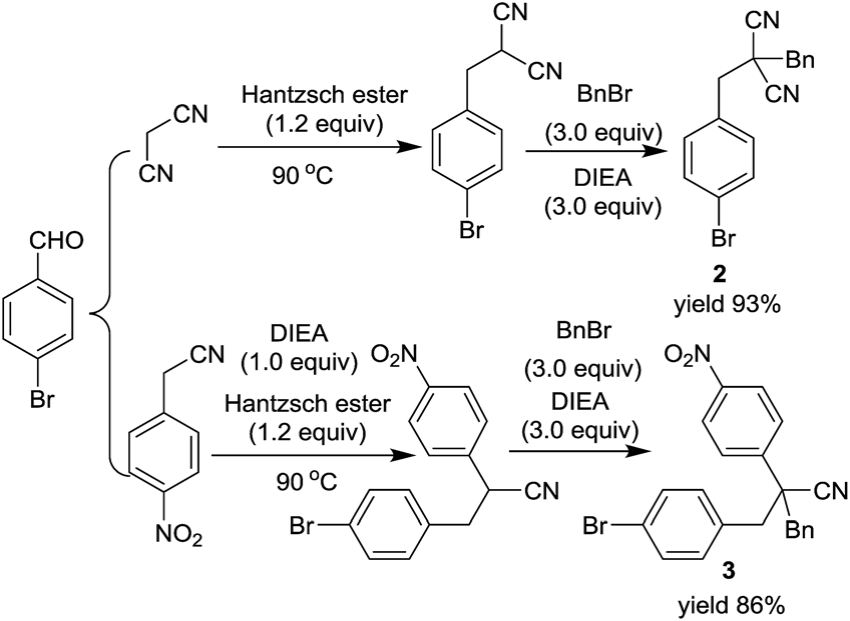
Solvent-free one-pot two step RRC/A reaction.

In conclusion, an efficient one pot solvent-free four components RRC/A process for the synthesis of 2,2-dialkylated cyano esters has been described by the reaction of commercially available aldehydes, ethyl cyanacetates, alkyl halides and Hantzsch ester. The designed RRC/A products could be obtained with moderate to high yields. And a solvent free one pot two step RRC/A process has also been developed for the preparation of 2,2-dialkylated malononitriles and 2,2-dialkylated 4-nitrophenyl acetonitriles. A series of malononitrile and 4-nitrophenyl acetonitrile derivatives could be prepared by these two step RRC/A methods. Compared with the first RRC/A method, the diversity of the substrate needs to be further improvement in this work. Nevertheless, this method provided a weak base catalyzed one pot solvent free version of RRC/A reaction, which is a complement to the first one.

## Conflicts of interest

There are no conflicts to declare.

## Supplementary Material

RA-008-C8RA00326B-s001
