# Supplementary material for: A base promoted one pot solvent free version of the Ramachary reductive coupling/alkylation reaction for the synthesis of 2,2-disubstituted ethyl cyanoacetates
Source: RSC Adv. 2018 Feb 28;8(16):8961–4. doi: 10.1039/c8ra00326b (PMC9078616; doi:10.1039/c8ra00326b)

Electronic Supplementary Material (ESI) for RSC Advances.
This journal is © The Royal Society of Chemistry 2018

Supporting Information

Base promoted one pot solvent free version of Ramachary reductive coupling/alkylation reaction for the synthesis of 2,2-disubstituted ethyl cyanoacetates

Guangyou Jiang[a#], Min Liu[a#], Dongmei Fang[b], Ping Tan[a], Min Huang[a], Taiping Zhou[a], Zhenju Jiang[a], Zhihong Xu*[a], Zhouyu Wang*[a]

[a] Department of Chemistry, Xihua University, Chengdu, 610039, China. E-mail: zhouyuwang77@163.com, xzh1966@163.com
[b] Chengdu Institute of Biology, Chinese Academy of Sciences, Chengdu, 610041, China.
[#]These authors contributed equally to this work and should be considered co-first authors.
Fax: +86-028-8772-3006; Tel: +86-028-8772-9463

General Methods: All starting materials were of the commercially available (analytical grade) and used without further purification. All the solvents are used after redistillation. Reactions were monitored by thin layer chromatography using silica gel HSGF254 plates. Flash chromatography was performed using silica gel HG/T2354-92. Melting points were measured with SGW X-4 melting point apparatus. ^{1}H NMR (400 MHz) spectra were recorded in $CDCl_3$ or DMSO. ^{1}H NMR chemical shifts are reported in ppm (δ) relative to tetramethylsilane (TMS) with the solvent resonance employed as the internal standard ($CDCl_3$, δ = 7.26 ppm；DMSO, δ = 2.50 ppm). Data are reported as follows: chemical shift, multiplicity (s = singlet, d = doublet, q = quartet, m = multiplet), coupling constants (Hz) and integration.^{13}C NMR chemical shifts are reported in ppm from tetramethylsilane (TMS) with the solvent resonance as the internal standard ($CDCl_3$, 77.0 ppm; DMSO, 39.5 ppm). Chemical yields refer to pure isolated substances. ESIMS spectra were recorded on BioTOF Q. All products were prepared according to the general procedure. Except for **1f** and **1i**, all the products are unknown compounds.

General experimental procedure for preparing 2, 2-disubstituted ethyl cyanoacetates: Under solvent-free conditions, the mixture of aldehydes (0.25 mmol), ethyl 2-cyanoacetate (0.30 mmol), benzyl bromide (0.75 mmol), DIEA (0.50 mmol) and Hantzsch esters (0.30 mmol) was stirred at 90 ℃ for 2 h. After the completion of the K/H/A process and the reaction mixtures were cooled to room temperature. Then the residue was purified through column chromatograph on silica gel to give the pure products**.**

General experimental procedure for preparing 2, 2-disubstituted 2-(4-nitrophenyl)-acetonitrile: Under solvent-free conditions, the mixture of aldehydes (0.25 mmol), 2-(4-nitrophenyl)-acetonitrile (0.25 mmol), DIEA (0.25 mmol) and Hantzsch esters (0.30 mmol) was stirred at 90 ℃ for 2 h. After the completion of the K/H process and the reaction mixtures were cooled to room temperature. Without any work up process, the benzyl bromide (0.75 mmol) with DIEA (0.75 mmol) were added and the stirring was maintained at 90 ℃ for 2 h. The reaction mixtures were cooled to room temperature. The residue was purified through column chromatograph（Petroleum ether : EtOAc ，80:1→15:1） onto silica gel to give the crude products. Finally, all the crude products are purified through the silica gel plate (200 mm × 200 mm, Petroleum ether : CH_2Cl_2 = 1: 1).

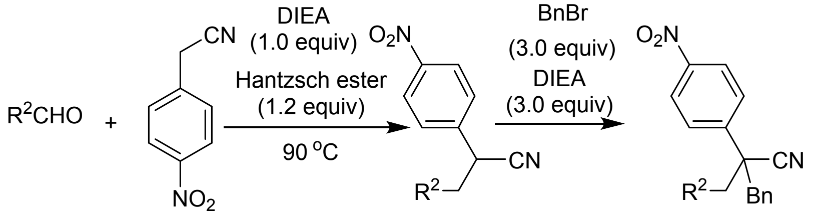

Entry	R^2	Yield (%)
1	C_6H_5	90
2	4-BrC_6H_4	86
3	4-ClC_6H_4	76
4	4-FC_6H_4	85
5	4-MeC_6H_4	95
6	4-$MeOC_6H_4$	76
7	4-NCC_6H_4	88
8	1-Naphthyl	83
9	2-furyl	62

10	2-thienyl	68

General experimental procedure for preparing 2, 2-disubstituted malononitrile: Under solvent-free conditions, the mixture of aldehydes (0.25 mmol), malononitrile(0.25 mmol) and Hantzsch esters (0.30 mmol) was stirred at 90 ℃ for 2 h. After the completion of the self-catalyzed K/H process and the reaction mixtures were cooled to room temperature. Without any work up process, the benzyl bromide (0.75 mmol) with DIEA (0.75 mmol) were added and stirred at 90 °C for 2 h. The reaction mixtures were cooled to room temperature. The residue was purified through column chromatograph（Petroleum ether : EtOAc ，80:1→15:1） onto silica gel to give the crude products. Finally, all the crude products are purified through the silica gel plate (200 mm × 200 mm, Petroleum ether : CH_2Cl_2 = 1: 1).

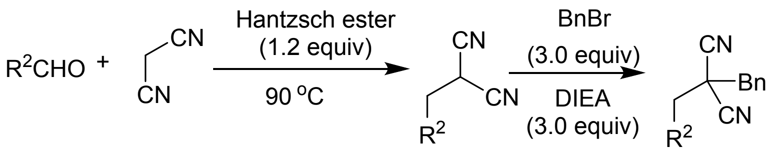

Entry	R^2	Yield (%)
1	C_6H_5	86
2	4-BrC_6H_4	93
3	4-ClC_6H_4	93
4	4-FC_6H_4	80
5	4-MeC_6H_4	88
6	4-MeOC_6H_4	94
7	4-NCC_6H_4	61
8	4-$NO_2C_6H_4$	80
9	1-Naphthyl	92
10	2-furyl	80
11	2-thienyl	90

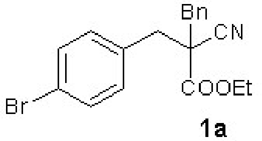

1a

ethyl 2-benzyl-3-(4-bromophenyl)-2-cyanopropanoate (1a): The crude mixture was purified by column chromatography using Petroleum ether /EtOAc (60/1→15/1) to yield **1a** as yellow liquid with 92% yield. ^{1}H NMR (400 MHz, $CDCl_3$) δ 7.47 (d, J = 8.32 Hz, 2H), 7.32 - 7.34 (m, 5H), 7.20 (d, J = 8.32 Hz, 2H), 4.03 (q, J = 7.12 Hz, 2H), 3.34, 3.32 (AB system, J_{AB} = 13.40 Hz, 2H), 3.13, 3.08 (AB system, J_{AB} = 13.44 Hz, 2H), 1.03 (t, J = 7.12 Hz, 3H). ^{13}C NMR (100 MHz, DMSO) δ 167.9, 149.4, 143.5, 134.9, 130.3, 128.9, 128.2, 118.6, 111.1, 109.4, 62.9, 52.0, 42.2, 35.5, 14.2. ESI HRMS exact mass calcd. for $(C_{19}H_{18}BrNO_2 + Na)^+$ requires m/z 394.04099, found m/z 394.04089.

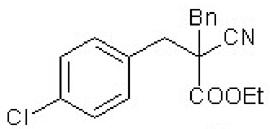

1b

ethyl 2-benzyl-3-(4-chlorophenyl)-2-cyanopropanoate (1b): The crude mixture was purified by column chromatography using Petroleum ether /EtOAc (60/1→15/1) to yield **1b** as yellow oily liquid with 96% yield. ^{1}H NMR (400 MHz, DMSO) δ 7.25 - 7.45 (m, 9 H), 4.04 (q, J = 7.12 Hz, 2H), 3.36, 3.33 (AB system, J_{AB} = 11.32 Hz, 2H), 3.27, 3.23 (AB system, J_{AB} = 13.56 Hz, 2H), 1.01 (t, J = 7.12 Hz, 3H). ^{13}C NMR (100 MHz, DMSO) δ168.1, 135.1, 135.0, 134.1, 133.0, 132.1, 130.3, 128.9, 128.1, 118.9, 62.8, 54.0, 42.8, 41.8, 14.1. ESI HRMS exact mass calcd. for $(C_{19}H_{18}ClNO_2 + Na)^+$ requires m/z 350.09168, found m/z 350.09172.

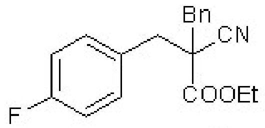

1c

ethyl 2-benzyl-2-cyano-3-(4-fluorophenyl)propanoate (1c): The crude mixture was purified by column chromatography using Petroleum ether /EtOAc (60/1→15/1) to yield **1c** as yellow liquid with 95% yield.^{1}H NMR (400 MHz, DMSO) δ 7.18 - 7.38 (m, 9H), 4.04 (q, J = 7.12 Hz, 2H), 3.37, 3.36 (AB system, J_{AB} = 11.52 Hz, 2H), 3.29, 3.25 (AB system, J_{AB} = 10.96 Hz, 2H), 1.01 (t, J = 7.12 Hz, 3H). ^{13}C NMR (100 MHz, DMSO) δ168.0, 163.4, 161.0, 135.1, 132.3, 131.3, 130.2, 128.9, 128.1, 118.8, 115.9, 62.8, 54.0, 42.6, 41.8, 14.1. ESI HRMS exact mass calcd. for $(C_{19}H_{18}FNO_2 + H)^+$ requires m/z 312.1394, found m/z 312.1382.

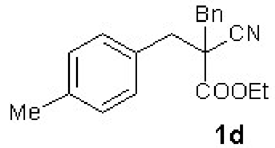

1d

ethyl 2-benzyl-2-cyano-3-p-tolylpropanoate (1d): The crude mixture was purified by column chromatography using Petroleum ether /EtOAc (60/1→15/1) to yield **1d** as white colorless liquid 95% yield. ^{1}H NMR

(400 MHz, DMSO) δ 7.13 - 7.38 (m, 9H), 4.04 (q, J = 7.12 Hz, 2H), 3.18 - 3.35 (m, 4H), 2.29 (s, 3H), 1.03 (t, J = 7.12 Hz, 3H). ^{13}C NMR (100 MHz, DMSO) δ168.1, 137.3, 135.1, 132.0, 130.3, 130.1, 129.5, 128.9, 128.1, 118.9, 62.7, 54.1, 42.8, 42.4, 21.1, 14.2. ESI HRMS exact mass calcd. for $(C_{20}H_{21}NO_2 + H)^+$ requires m/z 308.1644, found m/z 308.1647.

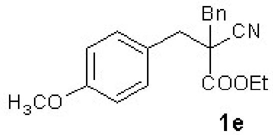

1e

ethyl 2-benzyl-2-cyano-3-(4-methoxyphenyl)propanoate (1e): The crude mixture was purified by column chromatography using Petroleum ether /EtOAc (60/1→15/1) to yield **1e** as colorless liquid with 73% yield. ^{1}H NMR (400 MHz, DMSO) δ7.26-7.36 (m, 5H), 7.20 (d, J = 8.64 Hz, 2H), 6.92 (d, J = 8.64 Hz, 2H), 4.06 (q, J = 7.08 Hz, 2H), 3.75 (s, 3H), 3.35, 3.31 (AB system, J_{AB} = 13.32 Hz, 2H), 3.24, 3.22 (AB system, J_{AB} = 14.00 Hz, 2H),, 1.02-1.05 (t, J = 7.12 Hz， 3H). ^{13}C NMR (100 MHz, DMSO) δ 168.2, 159.2, 135.2, 131.4, 130.2, 128.9, 128.1, 126.9, 119.0, 114.3, 62.7, 55.5, 54.2, 42.7, 42.1, 14.2. ESI HRMS exact mass calcd. for $(C_{20}H_{21}NO_3 + H)^+$ requires m/z 324.1594, found m/z 324.1593.

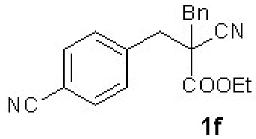

1f

ethyl 2-benzyl-2-cyano-3-(4-cyanophenyl)propanoate (1f): The crude mixture was purified by column chromatography using Petroleum ether /EtOAc (60/1→15/1) to yield **1f** as colorless liquid with 24% yield.^{1}H NMR (400 MHz, DMSO) δ 7.31 - 7.39 (m, 5H), 7.25 - 7.31 (m, 4H), 4.03 (q, J = 7.12 Hz, 2H), 3.37, 3.34 (AB system, J_{AB} = 13.56 Hz, 2H), 3.27, 3.24 (AB system, J_{AB} = 13.56 Hz, 2H), 1.01 (t, J = 7.12 Hz, 3H). See reference 16.

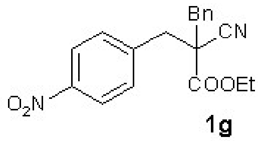

1g

ethyl 2-benzyl-2-cyano-3-(4-nitrophenyl)propanoate (1g): The crude mixture was purified by column chromatography using Petroleum ether /EtOAc (60/1→15/1) to yield **1g** as white solid with 76% yield. ^{1}H NMR (400 MHz, $CDCl_3$) δ 8.22 (d, J = 8.72 Hz, 2H), 7.51 (d, J = 8.72 Hz, 2H), 7.31 - 7.40 (m, 5H), 4.03 – 4.08 (m, 2H), 3.48, 3.45 (AB system, J_{AB} = 13.40 Hz, 2H), 3.20, 3.16 (AB system, J_{AB} = 13.48 Hz, 2H), 1.03 (t, J = 7.16 Hz, 3H). ^{13}C NMR (100 MHz, DMSO) δ167.8, 147.6, 143.0, 134.8, 131.7, 130.3, 129.0, 128.2, 124.0, 118.5, 63.1, 53.5, 42.7, 41.8, 14.1. ESI HRMS exact mass calcd. for $(C_{19}H_{18}N_2O_4 + H)^+$ requires m/z 338.1194, found m/z 338.1124.

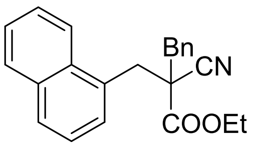

1h

2-benzyl-2-((naphthalen-5-yl)methyl)malononitrile (1h): The crude mixture was purified by column chromatography using Petroleum ether /EtOAc (60/1→15/1) to yield **1h** as colorless liquid with 92% yield.^{1}H NMR (400 MHz, $CDCl_3$) δ 8.23 (d, *J* =7.84 Hz, 1H), 7.90 - 7.97 (m, 2H), 7.37 - 7.60 (m, 4H), 7.31 - 7.35 (m,5H), 3.91 (q, *J* = 7.08 Hz, 2H), 3.87 (s, 2H), 3.52, 3.48 (AB system, J_{AB} = 13.52 Hz, 2H), 0.88 (t, *J* = 7.12 Hz, 3H). ^{13}C NMR (100 MHz, DMSO) δ168.3, 135.3, 134.0, 132.3, 131.6, 130.4, 129.0, 128.9, 128.5, 128.1, 126.4, 126.3, 125.7, 124.6, 119.0, 62.8, 53.4, 42.9, 38.4, 13.9. ESI HRMS exact mass calcd. for $(C_{23}H_{21}NO_2 + H)^+$ requires m/z 344.1604, found m/z 344.1602.

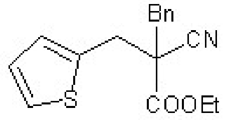

1i

ethyl 2-benzyl-2-cyano-3-(thiophen-2-yl)propanoate (1i): The crude mixture was purified by column chromatography using Petroleum ether /EtOAc (60/1→15/1) to yield **1i** as yellow liquid with 94% yield.^{1}H NMR (400 MHz, DMSO) δ 7.47 (d, *J* = 1.28 Hz, 1H), 7.33 - 7.46 (m, 3H), 7.25 - 7.27 (m, 2H), 7.00 - 7.03 (m, 2H), 4.09 (q, *J* = 7.12 Hz, 2H), 3.60, 3.53 (AB system, J_{AB} = 14.72 Hz, 2H), 3.35 (s, 2H), 1.07 (t, *J* = 7.12 Hz, 3H).

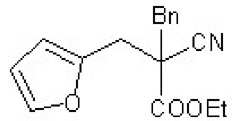

1j

ethyl 2-benzyl-2-cyano-3-(furan-2-yl)propanoate (1j): The crude mixture was purified by column chromatography using Petroleum ether /EtOAc (60/1→15/1) to yield **1j** as colorless liquid with 88% yield.^{1}H NMR (400 MHz, DMSO) δ 7.61 (d, *J* = 1.04 Hz, 1H), 7.26 - 7.39 (m, 6H), 6.43 (d, *J* = 5.0 Hz, 1H), 6.30 (d, *J* = 3.16 Hz, 1H), 4.12 (q, *J* = 7.12 Hz, 2H), 3.22 - 3.41 (m, 4H), 1.10 (t, *J* = 7.12 Hz, 3H). ^{13}C NMR (100 MHz, DMSO) δ167.8, 149.4, 143.5, 134.9, 130.3, 128.9, 128.2, 118.6, 111.1, 109.4, 62.9, 52.0, 42.2, 35.5, 14.1. ESI HRMS exact mass calcd. for $(C_{17}H_{17}NO_3 + H)^+$ requires m/z 284.1281, found m/z 284.1278.

References

1.R. Lopez and C. Palomo, *Angew. Chem. Int. Ed.*, 2015, **54**, 13170-13184.

2.C. Guyon, M. C. Duclos, M. Sutter, E. Metay and M. Lemaire, *Org Biomol Chem*, 2015, **13**, 7067-7075.

3.C. Zheng and S.-L. You, *Chem. Soc. Rev.*, 2012, **41**, 2498-2518.

4.Y.-Q. Yang and Z. Lu, *Chin. J. Chem.*, 2014, **32**, 650-653.
5.M. Rueping, J. Dufour and F. R. Schoepke, *Green Chemistry*, 2011, **13**, 1084-1105.
6.D. B. Ramachary and S. Jain, *Org. Biomol. Chem*, 2011, **9**, 1277-1300.
7.T. Wang, X.-G. Huang, J. Liu, B. Li, J.-J. Wu, K.-X. Chen, W.-L. Zhu, X.-Y. Xu and B.-B. Zeng, *Synlett*, 2010, 1351-1354.
8.D. B. Ramachary, R. Mondal and C. Venkaiah, *Org. Biomol. Chem*, 2010, **8**, 321-325.
9.D. B. Ramachary and M. Kishor, *Org. Biomol. Chem*, 2008, **6**, 4176-4187.
10.S. Kumar and K. K. Kapoor, *Synlett*, 2007, 2809-2814.
11.Y. Huang, *Synlett*, 2007, 2304-2305.
12.S. J. Connon, *Org. Biomol. Chem*, 2007, **5**, 3407-3417.
13.D. B. Ramachary, M. Kishor and G. B. Reddy, *Org. Biomol. Chem*, 2006, **4**, 1641-1646.
14.T. He, R. Shi, Y. Gong, G. Jiang, M. Liu, S. Qian and Z. Wang, *Synlett*, 2016, **27**, 1864-1869.
15.D. B. Ramachary, M. Kishor and K. Ramakumar, *Tetrahedron Lett.*, 2006, **47**, 651-656.
16.D. Eswararao, M. Bhaskar, O'. Steven, T. Tell, M. John A., *J. Am. Chem. Soc.*, 2013, **135**, 10934-10937.

^{1}H and ^{13}C NMR Spectra:

The ^{1}H NMR Spectra of 1a

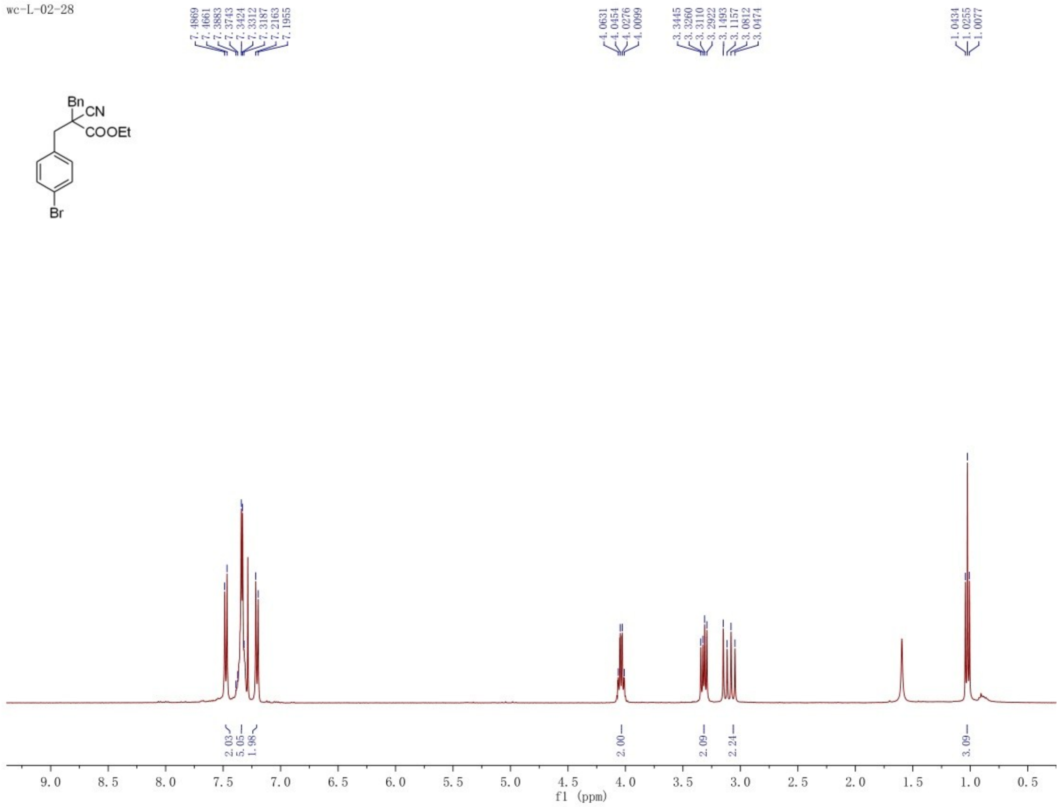

The ^{13}C NMR Spectra of 1a

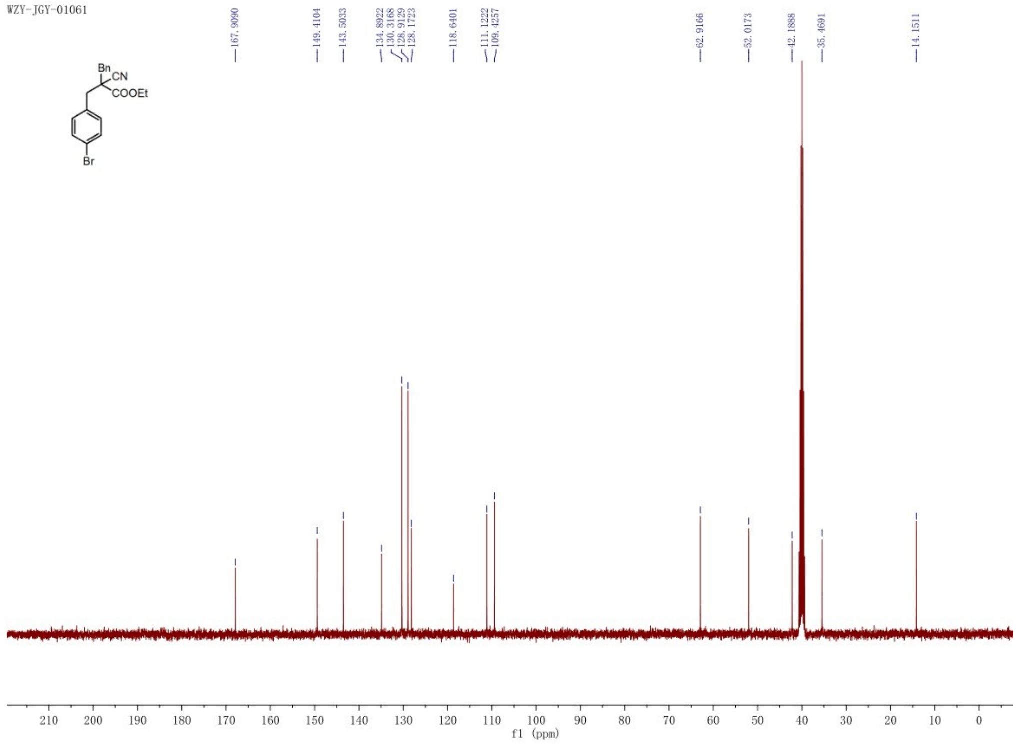

The [1] H NMR Spectra of 1b

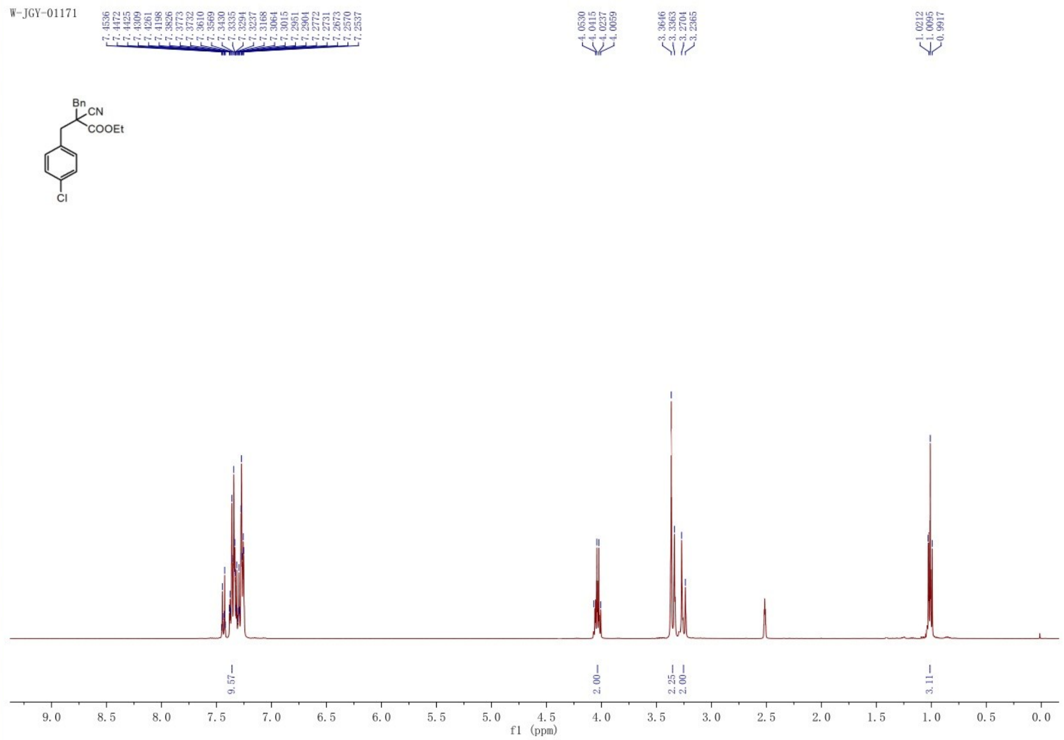

The [13] C NMR Spectra of 1b

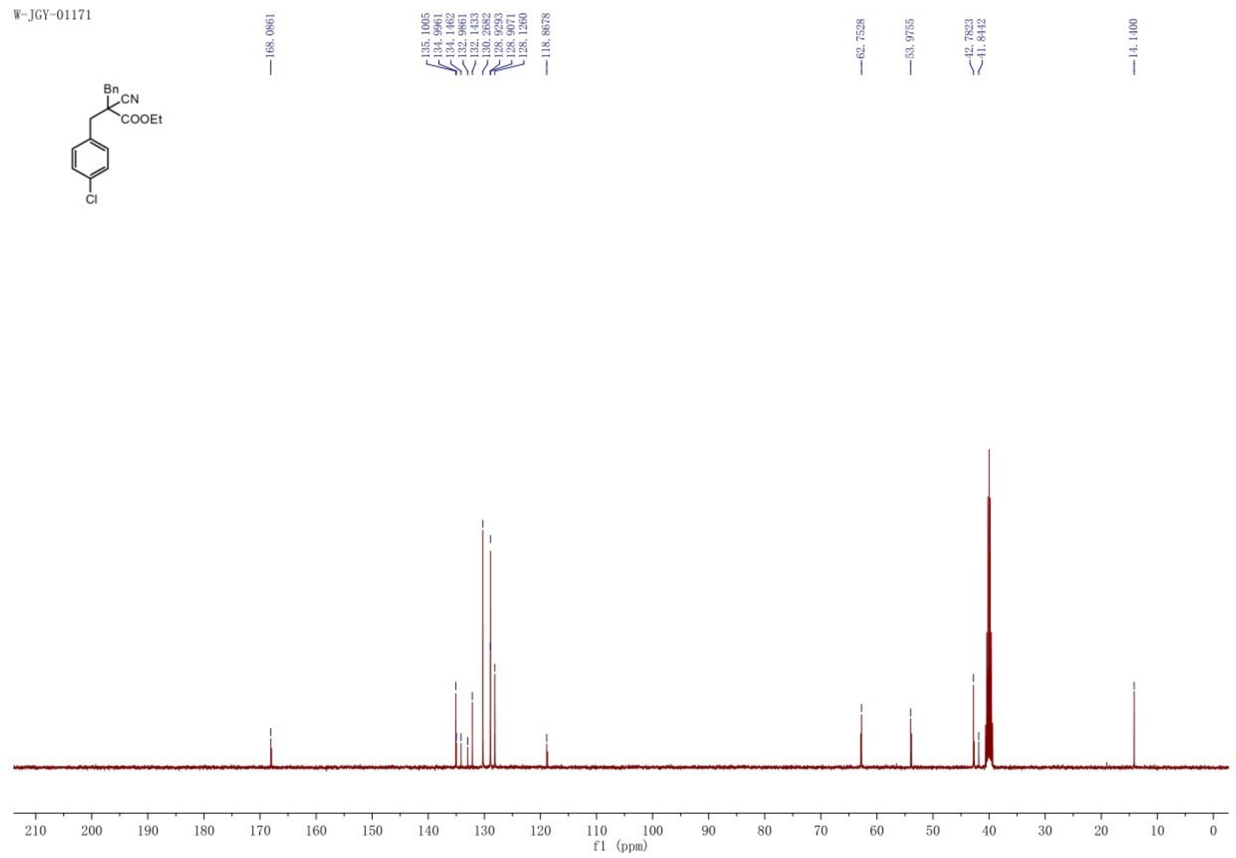

The ^{1}H NMR Spectra of 1c

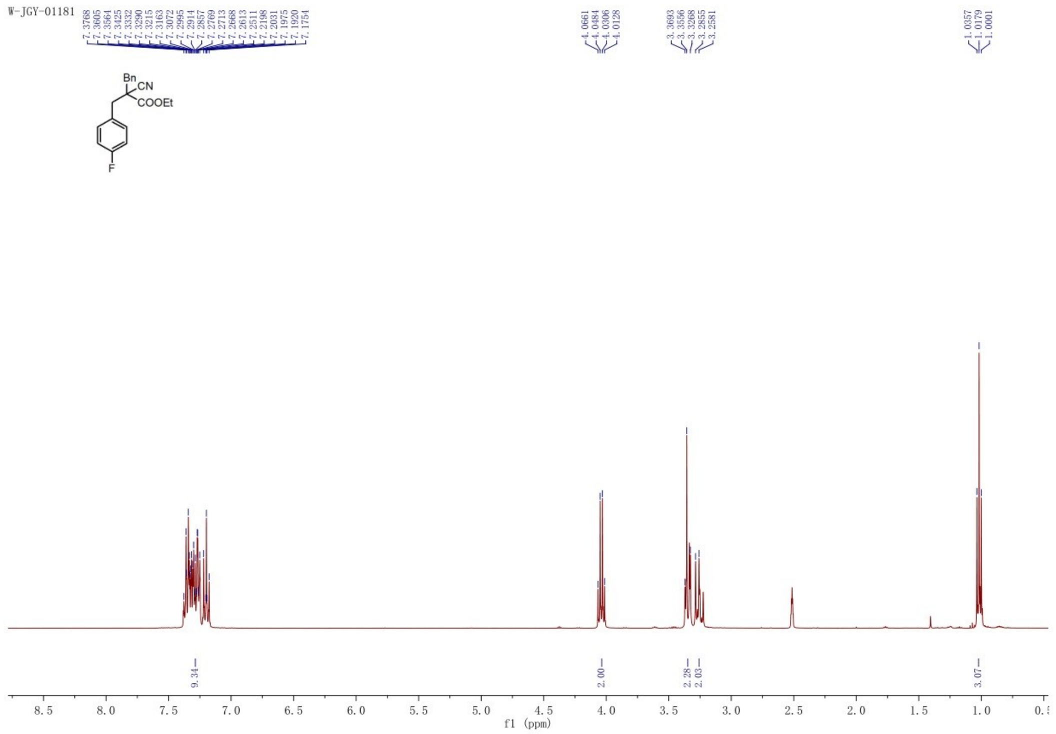

The ^{13}C NMR Spectra of 1c

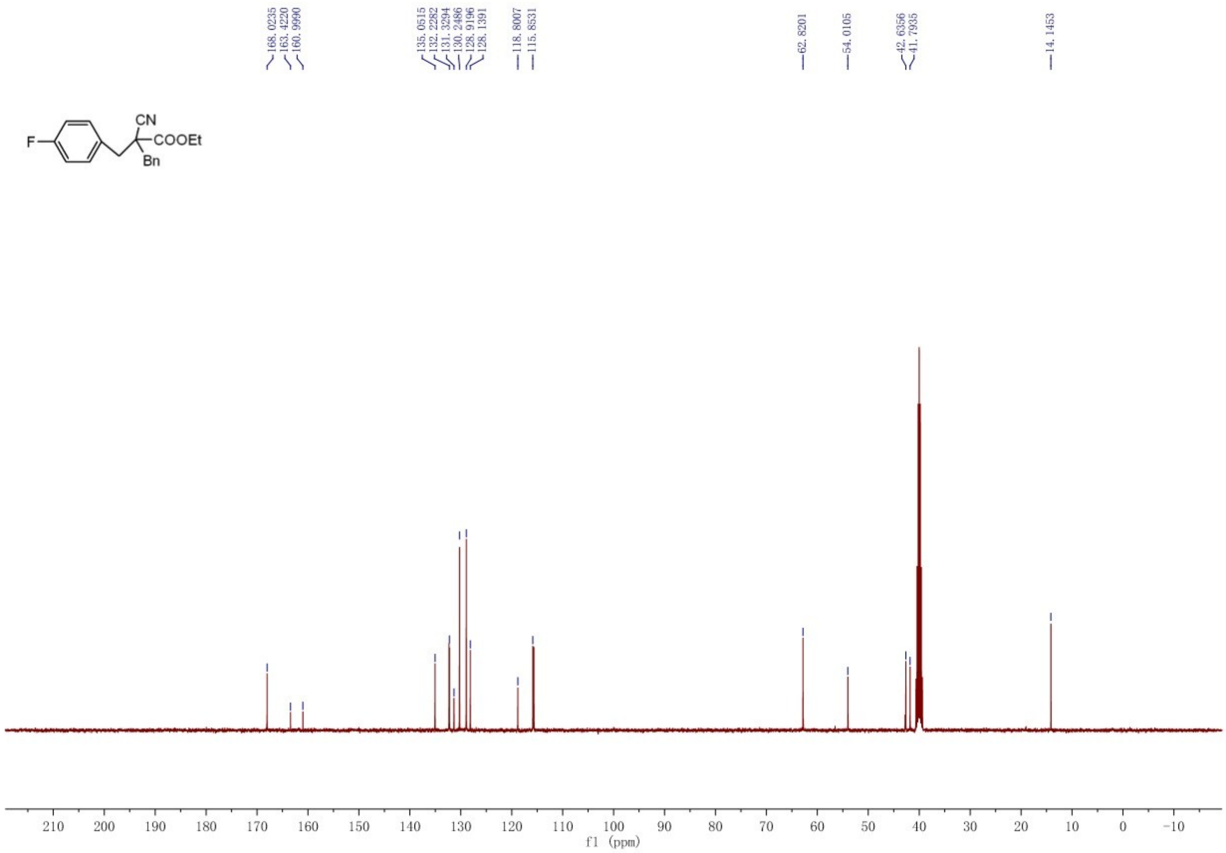

The [1] H NMR Spectra of 1d

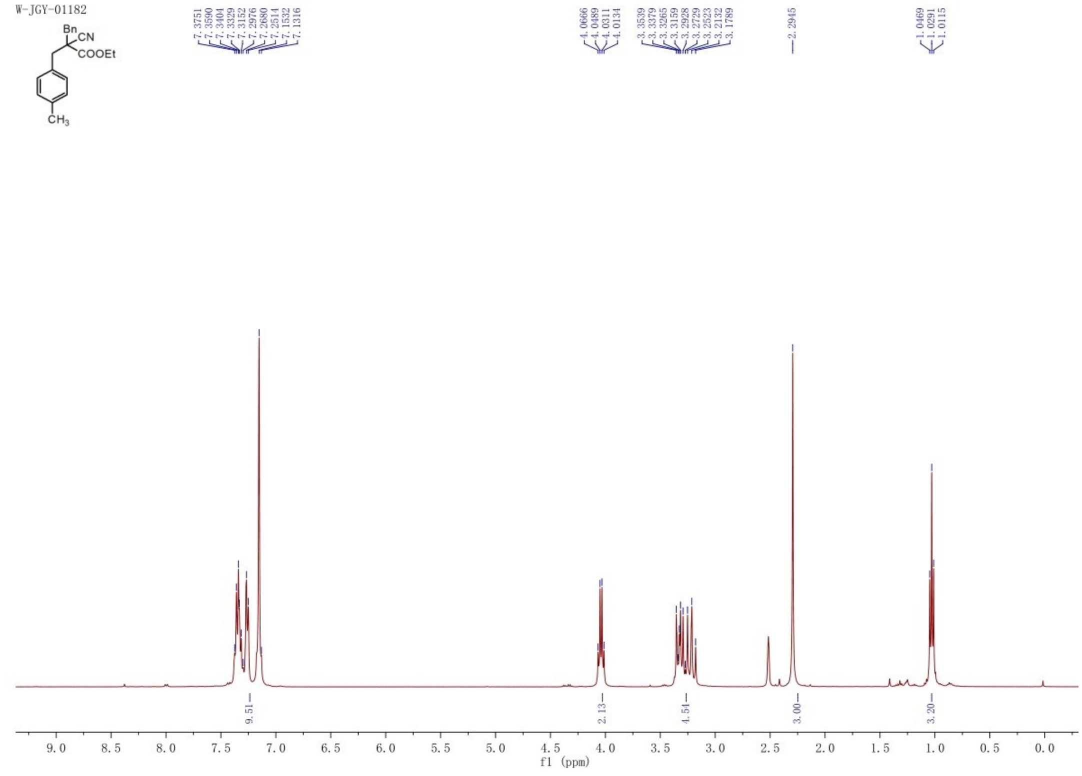

The ^{13}C NMR Spectra of 1d

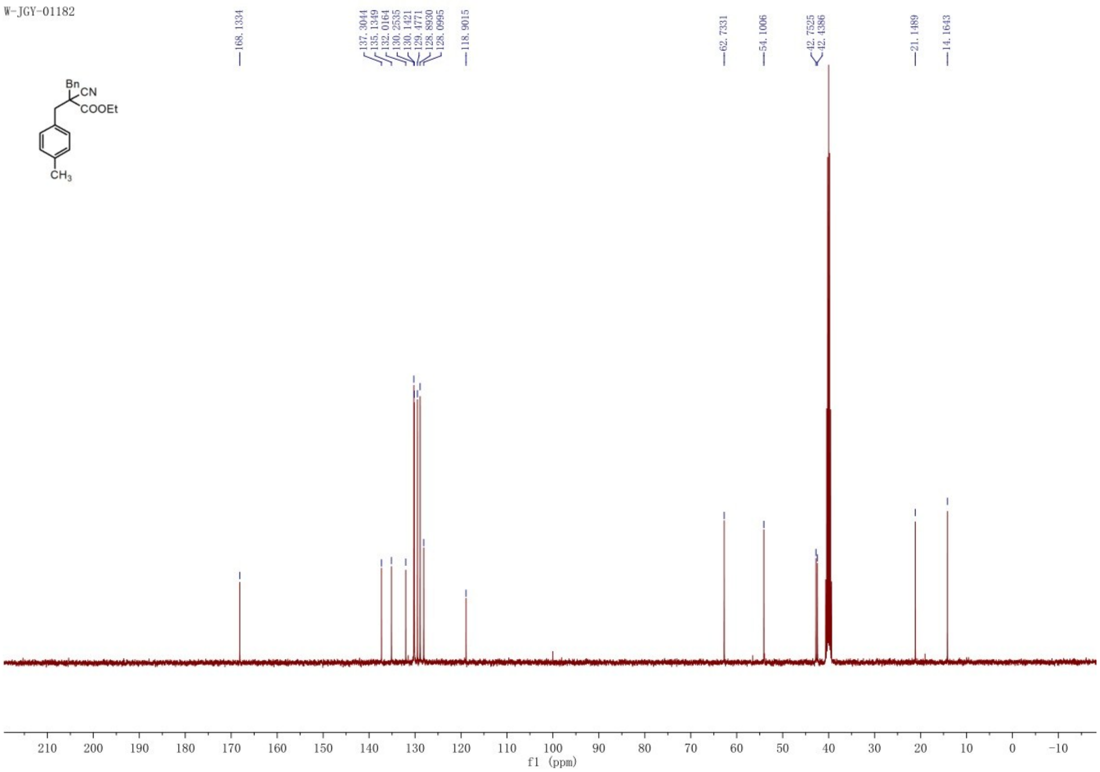

The ^{1}H NMR Spectra of 1e

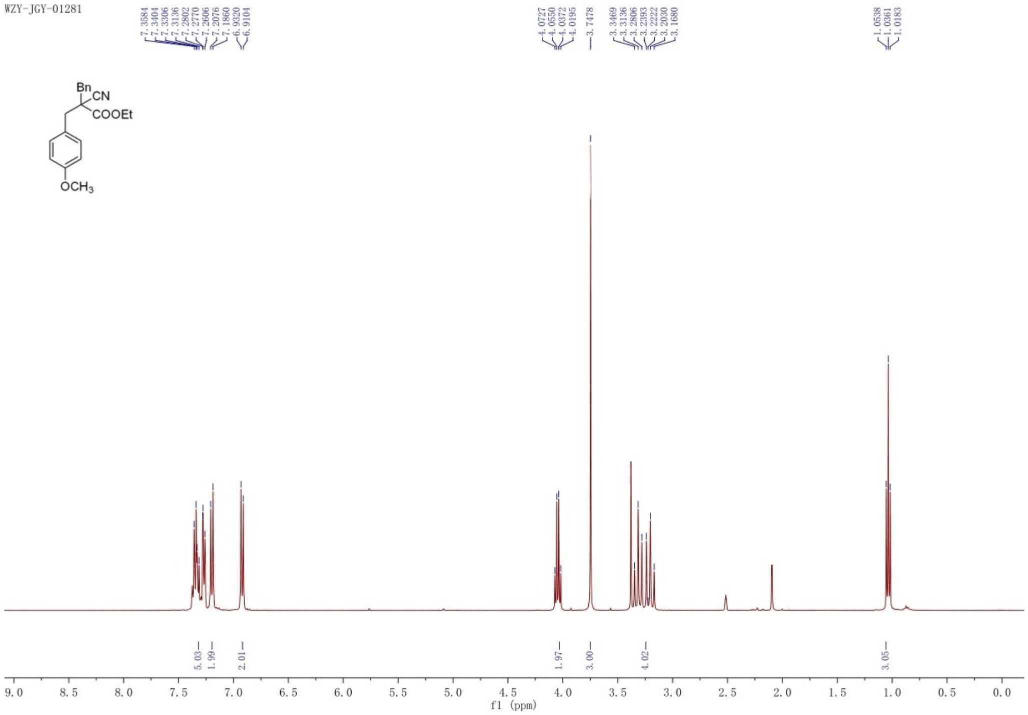

The ^{13}C NMR Spectra of 1e

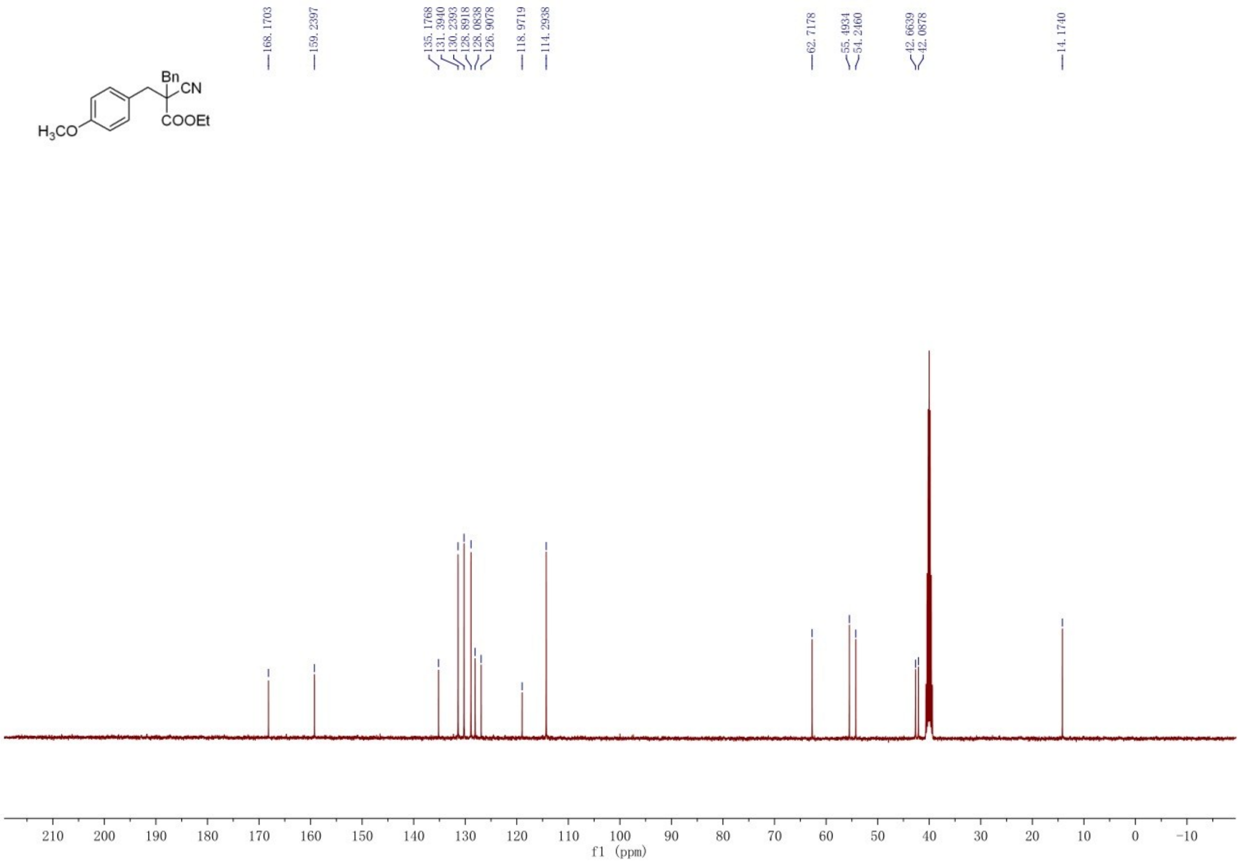

The 1 H NMR Spectra of 1f

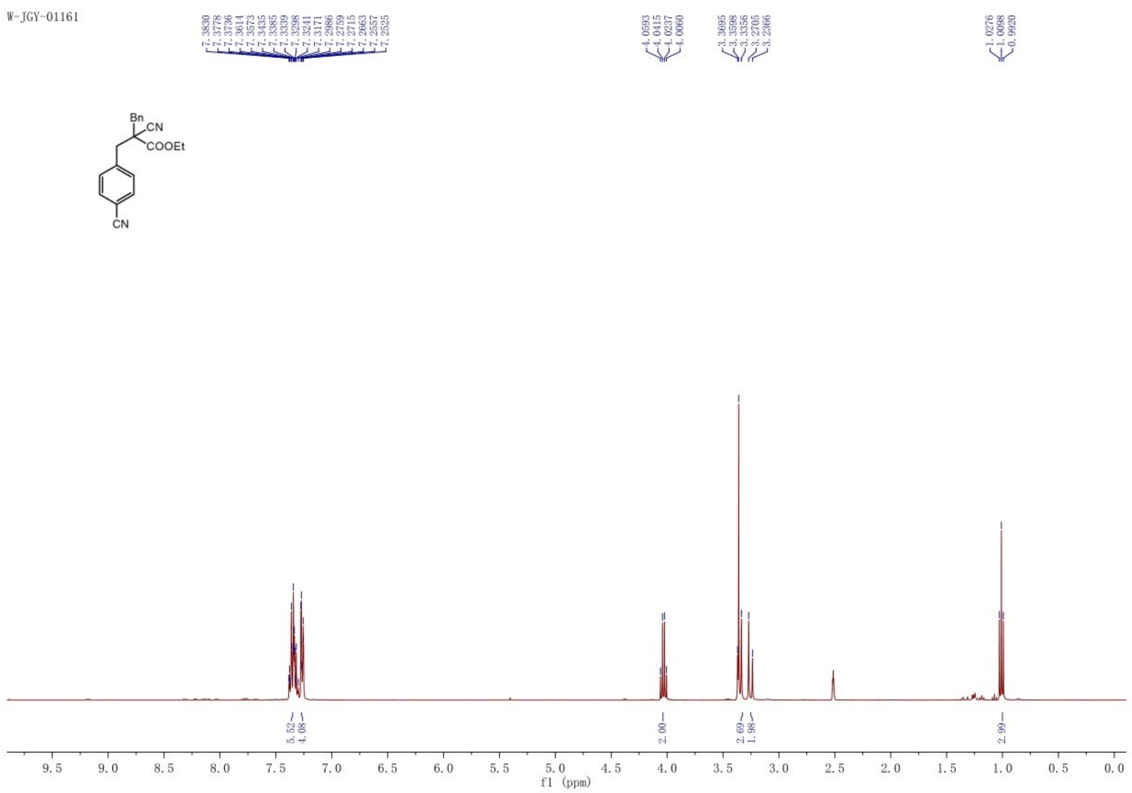

The ^{1}H NMR Spectra of 1g

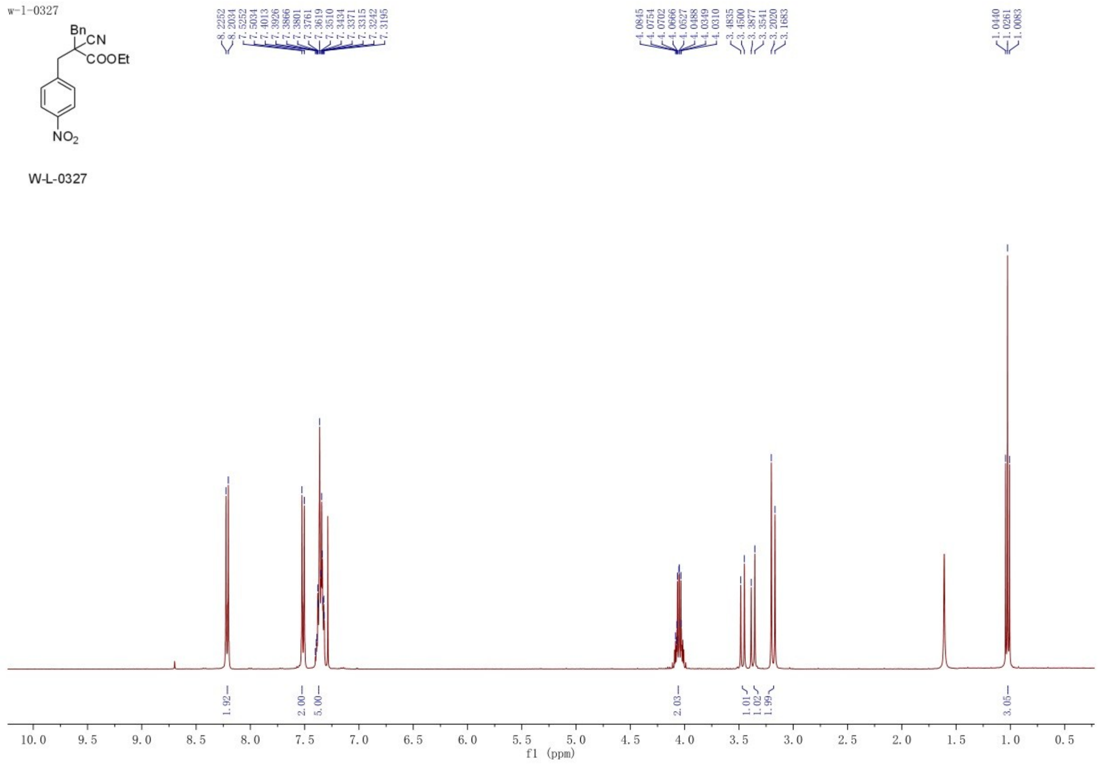

The ^{13}C NMR Spectra of 1g

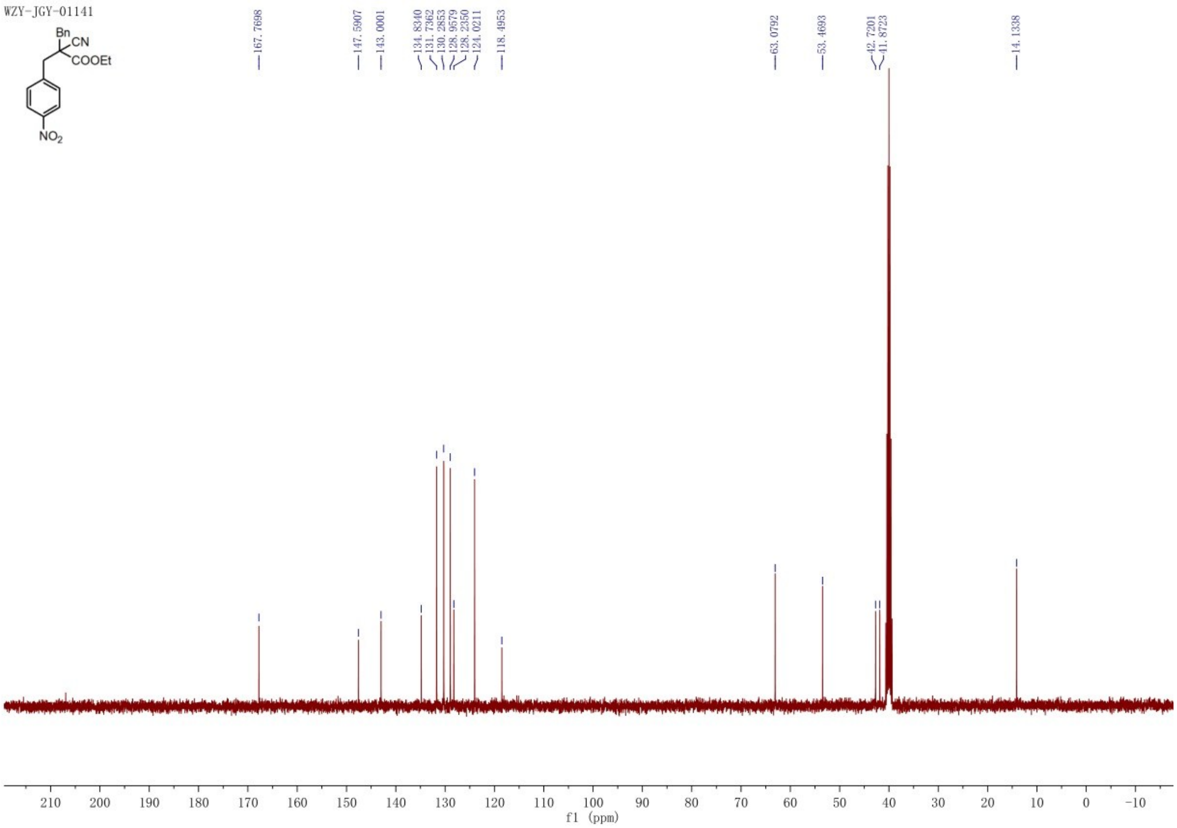

The [1] H NMR Spectra of 1h

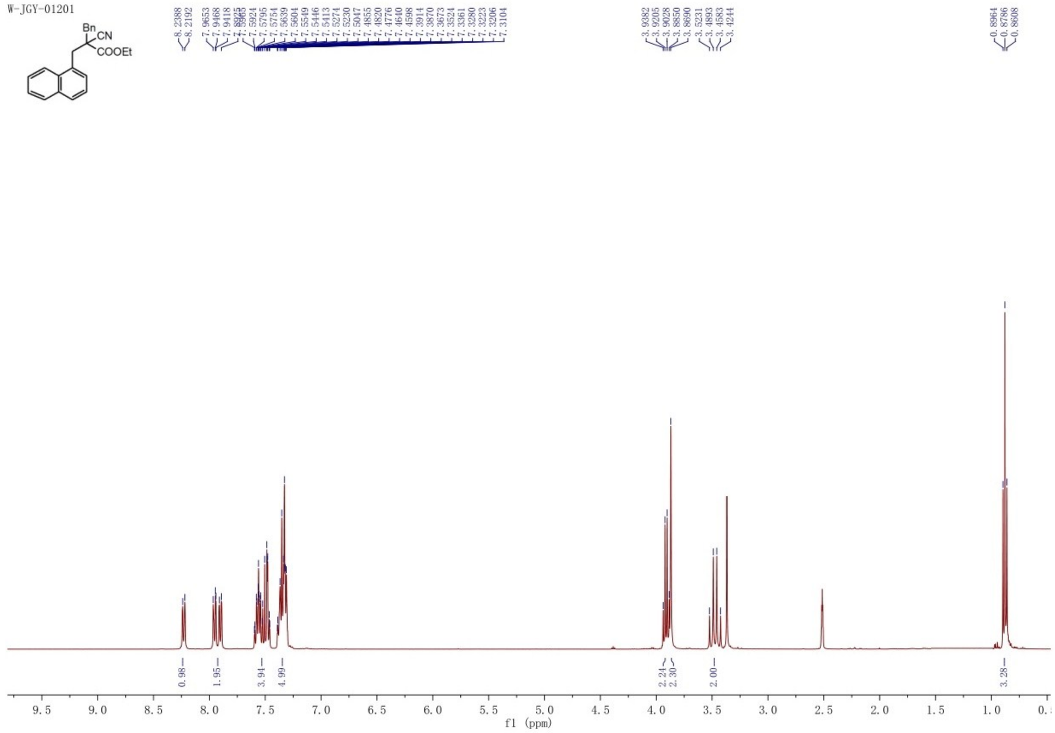

The [13] C NMR Spectra of 1h

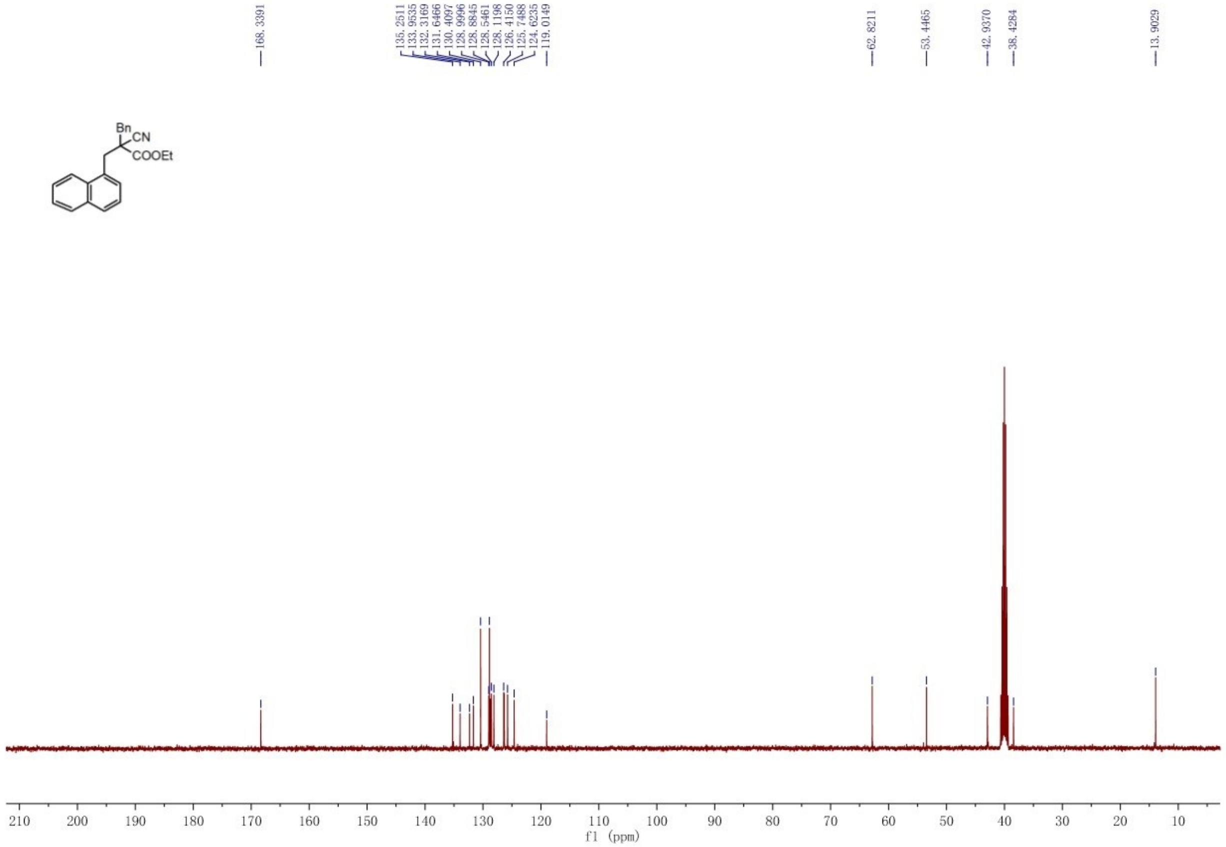

The [1] H NMR Spectra of 1i

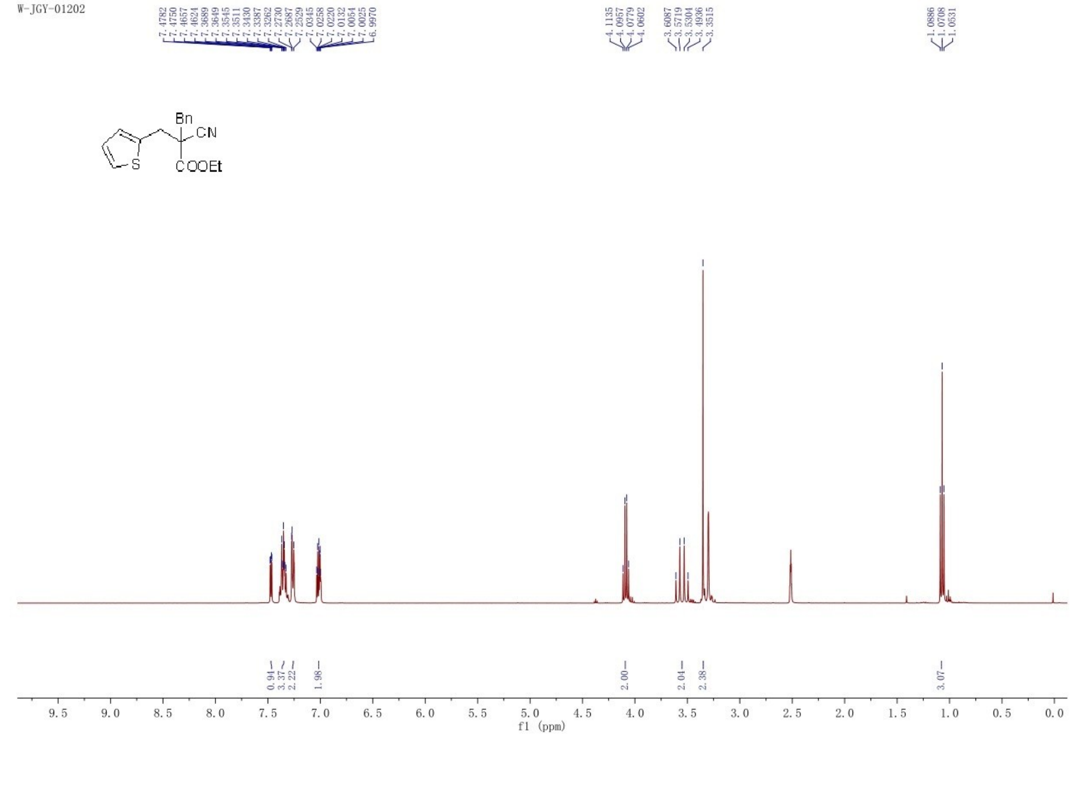

The [1] H NMR Spectra of 1j

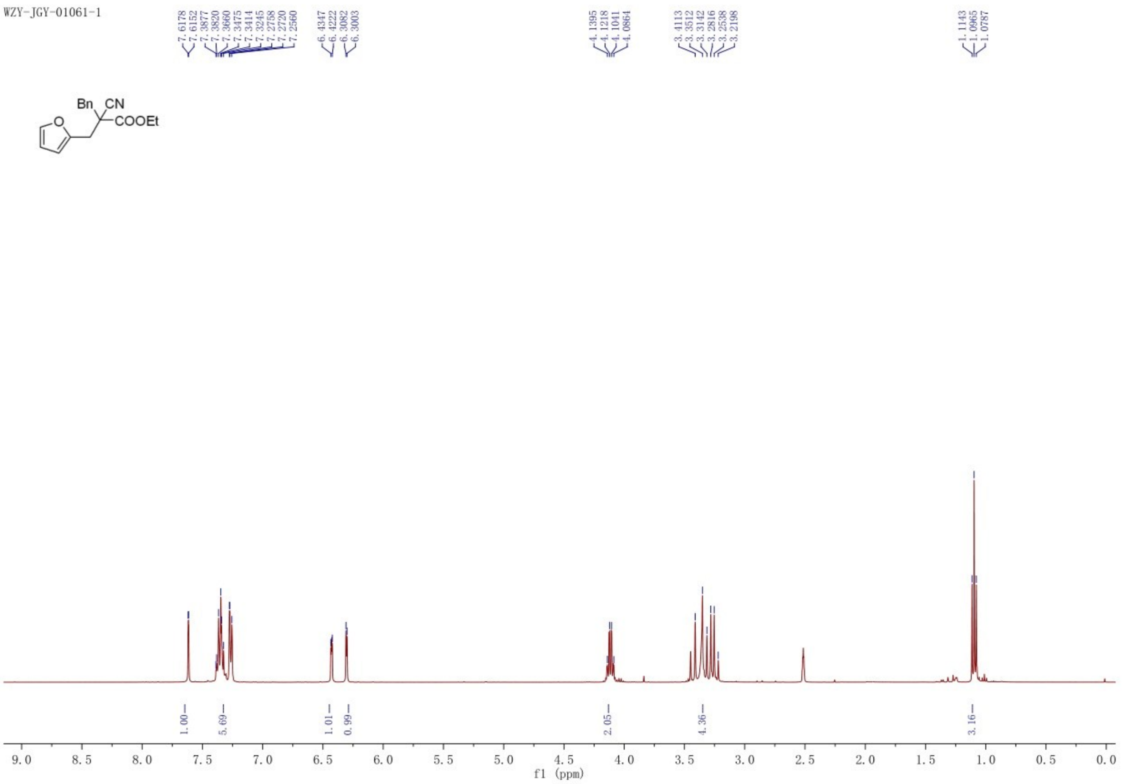

The [13] C NMR Spectra of 1j

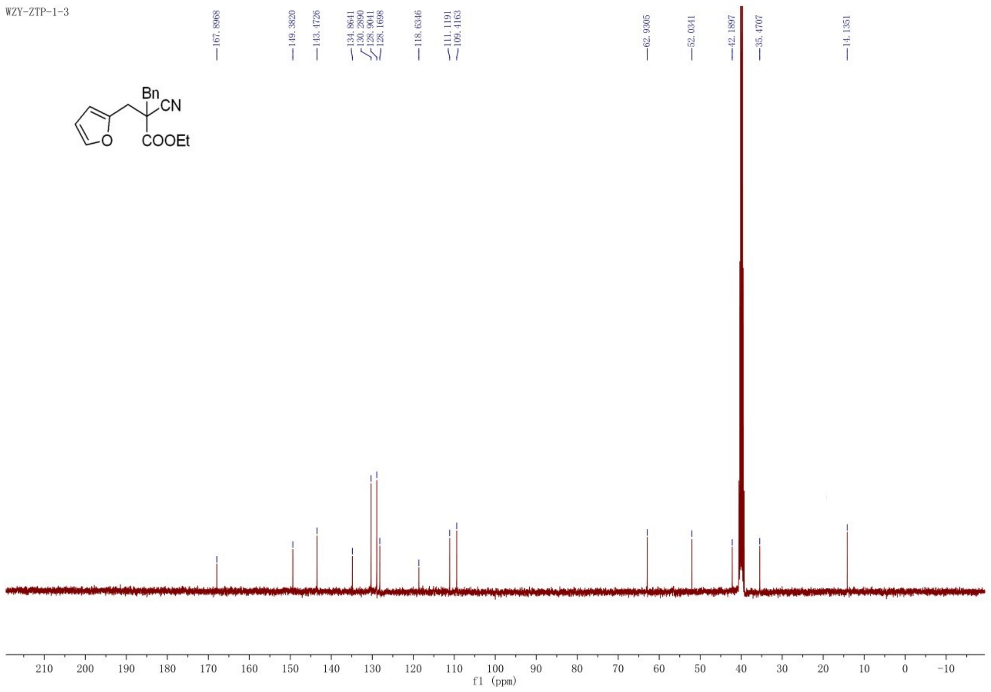

Supplement: RA-008-C8RA00326B-s001 [file RA-008-C8RA00326B-s001.pdf]
